# A Role for Neuropeptide S in Alcohol and Cocaine Seeking

**DOI:** 10.3390/ph15070800

**Published:** 2022-06-27

**Authors:** Nazzareno Cannella, Anna Maria Borruto, Michele Petrella, Maria Vittoria Micioni Di Bonaventura, Laura Soverchia, Carlo Cifani, Sara De Carlo, Esi Domi, Massimo Ubaldi

**Affiliations:** School of Pharmacy, University of Camerino, Via Madonna delle Carceri 9, 62032 Camerino, Italy; nazzareno.cannella@unicam.it (N.C.); annamaria.borruto@zi-mannheim.de (A.M.B.); michele.petrella@liu.se (M.P.); mariavittoria.micioni@unicam.it (M.V.M.D.B.); laura.soverchia@unicam.it (L.S.); carlo.cifani@unicam.it (C.C.); sara.decarlo@unicam.it (S.D.C.); esi.domi@unicam.it (E.D.)

**Keywords:** alcohol, cocaine, relapse, stress, anxiety, arousal

## Abstract

The neuropeptide S (NPS) is the endogenous ligand of the NPS receptor (NPSR). The NPSR is widely expressed in brain regions that process emotional and affective behavior. NPS possesses a unique physio-pharmacological profile, being anxiolytic and promoting arousal at the same time. Intracerebroventricular NPS decreased alcohol consumption in alcohol-preferring rats with no effect in non-preferring control animals. This outcome is most probably linked to the anxiolytic properties of NPS, since alcohol preference is often associated with high levels of basal anxiety and intense stress-reactivity. In addition, NPSR mRNA was overexpressed during ethanol withdrawal and the anxiolytic-like effects of NPS were increased in rodents with a history of alcohol dependence. In line with these preclinical findings, a polymorphism of the NPSR gene was associated with anxiety traits contributing to alcohol use disorders in humans. NPS also potentiated the reinstatement of cocaine and ethanol seeking induced by drug-paired environmental stimuli and the blockade of NPSR reduced reinstatement of cocaine-seeking. Altogether, the work conducted so far indicates the NPS/NPSR system as a potential target to develop new treatments for alcohol and cocaine abuse. An NPSR agonist would be indicated to help individuals to quit alcohol consumption and to alleviate withdrawal syndrome, while NPSR antagonists would be indicated to prevent relapse to alcohol- and cocaine-seeking behavior.

## 1. Introduction

The neuropeptide S (NPS) is a 20 amino acid neurotransmitter expressed by small clusters of neurons located within the parabrachial area, the peri-locus coeruleus (LC) and the sensory trigeminal nucleus [1]. The NPS receptor (NPSR) is expressed in brain nuclei such as the hypothalamus, hippocampus, amygdala and other limbic areas playing a role in motivated behavior [1,2]. Preclinical studies showed that NPS evokes a robust anxiolytic activity when administered centrally. These effects of NPS were assessed in several behavioral tests, in which NPS increased the time spent in the light area in the light–dark test, increased the number of the entries in the central zone of open field (OF) test, although this effect could be secondary to the increased locomotion, and increased the time spent in the open arms in the elevated plus maze (EPM) test [3,4]. A functional polymorphism of the NPSR gene was also associated with anxiety in humans [5]. NPS reduced the conditioned fear response, social avoidance and promoted fear extinction in rodents with mechanisms involving GABAergic pathways in the lateral and basolateral amygdala (LA, BLA) [6,7]. Additional studies showed that NPS injection into the endopiriform nucleus (EPN) reduced freezing and risk assessment behavior, suggesting that an NPS-mediated circuit comprising the EPN and BLA is involved in the processing of contextual fear memories [8]. On the other hand, studies on humans have led to apparently contradictive results. It has been found that a genetic variant of the human NPSR gene results in a functional boost of the NPSR, increasing the sensitivity to the agonist about tenfold [9]. This variant consists of a A > T polymorphism that leads to a change in one amino acid (Asn > Ile) of the NPSR protein. Noteworthy, individuals with the T allele showed a more conspicuous fear reaction to stimuli paired with painful electric stimulus then the individuals carrying the A allele. Collectively, these data indicate that the NPS system can be linked with a distorted interpretation of fear stimuli and its dysregulation might be associated with panic disorder [10,11]. The amygdala seems to be involved in this effect as demonstrated by fMRI studies that indicated a significant association of the T allele with amygdala responsiveness to fear-paired stimuli [12,13]. To reconciliate the apparently opposite results of the preclinical and clinical studies, it has been suggested that the levels of NPS critically affect the modulation of arousal and anxiety [14]. Previous studies have demonstrated that NPS activates the hypothalamic–pituitary–adrenal (HPA) axis. The microinjection of NPS into the paraventricular nucleus of the hypothalamus (PVN) increased adrenocorticotropic hormone (ACTH), corticosterone plasma levels and elicited a significant reduction of palatable food intake [15,16]. Moreover, NPS treatment increased the release of corticotropin-releasing factor (CRF) and arginine–vasopressin in hypothalamic explants [17]. Thus, the sustained NPS activity of the T genotype in humans could provoke an intense increase in arousal that might cause a correspondent stimulation of the HPA axis, triggering the insurgence of panic disorder. The NPS/NPSR system interacts with numerous others neurotransmitter systems implicated in stress, arousal, sleep–wake cycle and ingestive behavior. Double-labeling confocal microscopy of rat hypothalamus demonstrated that axons containing NPS are adjacent to Hcrt-1/Ox-A-positive neurons, that also express NPSR, suggesting a functional relationship between the two systems. Consistently, the hypocretin-1/orexin-A (Hcrt-1/Ox-A) selective receptor OX1 antagonist SB334867 blocked the exacerbation of drug seeking induced by NPS [18,19]. Moreover, centrally administered NPS evoked c-Fos expression in Hcrt-1/Ox-A neurons of the lateral hypothalamus (LH), the perifornical area (PeF) and in the dorsomedial hypothalamic nucleus (DMH) [20,21]. The corticotropin-releasing factor (CRF) system is also implicated in the effects of NPS on drug seeking and arousal. NPS failed to prime reinstatement of cocaine seeking and to stimulate locomotor activity in CRF receptor 1 (CRF1) knockout mice. Accordingly, the blockage of the CRF1 receptor by the antagonist antalarmin in wild-type mice blocked the NPS-induced reinstatement of cocaine seeking and increased locomotor activity. Interestingly, CRF knockout mice responded to the anxiolytic effect of NPS that were not blocked by antalarmin, indicating that the CRF system does not mediate the role of NPS in anxiety [22]. Evidence of a direct interaction between the NPS and CRF systems was also reported by a study showing that restraint stress increased c-Fos expression in NPS-expressing brain stem neurons co-expressing CRF1 receptor [23]. NPS also interacts with the glutamatergic neurotransmission, as a study demonstrated an activation of glutamatergic neurons in the EPN [8] and glutamatergic neurons in LC and trigeminal nucleus co-express NPS [2]. Recently, a circuit that is activated by stress and involves NPS/NPSR, OX1 receptors, NK1 receptors, mGlu5 receptors and CB1 receptors has been described [24]. The interaction with the CRF, orexin and glutamatergic systems can account for the pro-arousal functions of the NPS system and its involvement in stress modulation. On the other hand, the anxiolytic effects of NPS could be mediated by its action on amygdaloid GABAergic activity [8]. It was also demonstrated that a cluster of central amygdala (CeA) GABAergic neurons projects to the brainstem nuclei that express NPS, and this circuit is involved in the retrieval of fear memories [25]. Similarly, a GABAergic neuronal ensemble in the CeA, identified as protein kinase C δ (PKCδ + neurons) GABAergic positive neurons, was found to be crucial in driving compulsive drinking in a subset of rats and regulating the fear response through their brainstem projections [26,27]. It would be interesting to assess whether these inhibitory neurons also co-express NPS. Anxiety behavior induced by nerve injury was relieved by NPS through the increase of GABA release in the amygdala [28]. However, in another work, the anxiolytic-like effect of NPS was blocked by SHA68, a NPSR antagonist, but not by the GABA-A receptor antagonist picrotoxin [29], suggesting that the interaction between the NPS and GABAergic system in mediating anxiety could occur by indirect pathways. A recent study links NPS to the activity of the ventrolateral preoptic nucleus (VLPO), an important brain area for non-rapid eye movement (NREM) sleep, through a GABAergic mechanism that could have an important role in the sleep/wake cycle [30]. The anxiolytic effect of NPS has also been associated with oxytocinergic (OXTergic) activity, which recently has shown to correlate positively with the magnitude of alcohol self-administration and anxiety-like behavior [31]. NPSR is considerably expressed in the OXT neurons of the PVN, where NPS activates these neurons. Notably, the blockade of the PVN OXTergic neurons prevented the anxiolytic-like effect of NPS [32]. The physio-pharmacological profile of NPS is somehow paradoxical, being characterized by apparently antipodal features. As described above, the activation of this system modulates the fear response and produces anxiolytic-like effects. However, it also displays a pro-arousal effect, reducing sleep, enhancing alertness, increasing locomotor activity and facilitating spatial memory [3,4,6,33,34]. Mice lacking the expression of the NPS precursor displayed reduced arousal and the impairment of long-term memory [35]. The precursor of the NPS gene is present and highly conserved in all vertebrates, with the only exception represented by fish [36]. This highly conserved nucleotide sequence of the NPS gene indicates that the peptide has been subjected to considerable evolutionary pressure, suggesting a critical functional role. One appealing possibility is that the NPS system has evolved its physiological characteristics to permit the organism to confront dangerous situations in which intense arousal and alertness, together with mitigated anxiety and fear, could be needed as an effective coping strategy. The ambivalent nature of NPS effects can also help to explain findings that associated the two variants (A/T) of NPSR polymorphism with alcohol use disorders (AUD) in clinical cohorts of men and women diagnosed with AUD. Interestingly, AUD was associated with the A allele in females and the T allele in males. As mentioned before, the T allele leads to a higher activity of NPSR [37]. Previous studies have shown that individuals carrying this allele have poor impulse control [38], which is a risk factor for drug abuse [39]. On the other hand, it is well known that affective disorders, such as anxiety and depression, are risk factors for alcohol abuse and females carrying the A allele were more vulnerable to anxiety disorders [37,40]. Therefore, a possible interpretation is that the higher alcohol consumption shown by A allele female carriers is linked to their lower NPSR activity with consequent higher levels of anxiety [37]. The pharmacological targeting of the NPS/NPSR system could lead to the development of novel drugs useful to treat various disorders including anxiety and drug abuse.

Stress plays a major role in drug abuse and, despite the well-characterized mechanism by which stress promotes drug abuse, there is no approved drug that targets the stress system. Thanks to its dual effect on stress, the NPS system is a potential target to develop drugs targeting the stress system to treat drug abuse. For this reason, this review will describe the preclinical data supporting the role for the NPS/NPSR system in addiction-related behaviors and the pharmacological approaches that could lead to future therapeutical treatments.

## 2. Neurobiology of NPS

**Figure 1:**
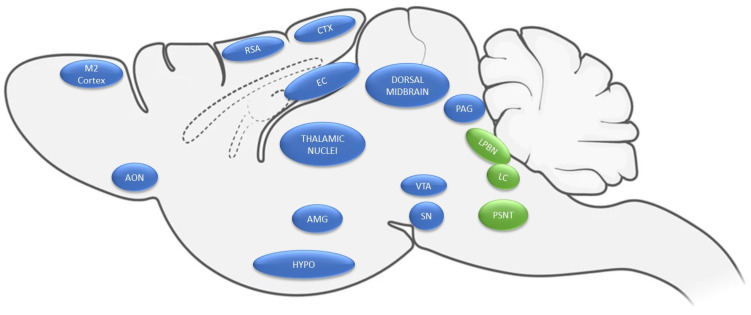
Schematic representation of NPS precursor and NPSR1 transcript distribution in the rat brain. Green circles (PSNT: principal sensory trigeminal nucleus, LPBN: lateral parabrachial nucleus and LC: peri-locus coeruleus) represent areas of NPS precursor mRNA expression, while blue circles (M2cortex: secondary motor cortex, AON: anterior olfactory nucleus, RSA: agranular retrosplenial cortex, CTX: cortex, EC: entorhinal cortex, thalamic nuclei, AMG: amygdala, HYPO: hypothalamus, dorsal midbrain; VTA: ventral tegmental area; SNC: substantia nigra, PAG: periaqueductal gray area) depict brain regions with NPSR1 transcript expression.

In the rat, NPS precursor mRNA is localized in a few discrete brain stem nuclei, showing the highest level of expression within the peri-LC, the lateral parabrachial nucleus (lPBN), and the principal trigeminal sensory nucleus; sparse expression has also been identified in the DMH and the amygdala [2,3]. Similar findings were reported in the mouse, although with some significant differences. Indeed, NPS expression in the mouse brain is even more restricted, being found only in the peri-LC and the lPBN (Kölliker–Fuse (KF)) nucleus of the lateral parabrachial area [1,41]. Noteworthy, NPS is often expressed together with other neurotransmitters and neuropeptides, indicating that NPS could be released in conjunction with them on the neural targets of the NPS-synthetizing neurons. Most of the peri-LC NPS-positive neurons express glutamatergic, but not GABAergic markers, suggesting that they co-release glutamate as neurotransmitter. Intriguingly, peri-LC NPS-expressing cells do not colocalize with the catecholaminergic marker tyrosine hydroxylase, indicating that these neurons represent a distinguishable non-noradrenergic (NAergic) cluster of cells in the peri-LC. Additionally, only few lateral peri-LC NPS-positive neurons express cholinergic markers, and they do not colocalize with CRF. Conversely, in the lPBN, many NPS-expressing neurons are also positive for CRF and galanin. Lastly, many NPS-positive neurons of the rat principal trigeminal sensory nucleus are believed to be of glutamatergic nature [1,2,3]. Recently, a study revealed the presence of NPS mRNA-expressing neurons in the human brainstem as well. Like in rodents, NPS is present in a cluster of neurons localized in the lPBN (spanning from the medial to the lateral subregions, including the KF nucleus); however, only few NPS-positive neurons were found in the human LC area, suggesting the presence of marked regional differences in NPS expression sites between the rodent and human brain [42]. In contrast to the focal localization of NPS, the pattern of expression of NPSR is much more dispersed within the rodent brain, being found in cortical regions, thalamic nuclei, the amygdala complex, hypothalamic regions and in the midbrain [2,41,43]. A schematic representation of NPS precursor and NPSR1 transcript distribution in the rat brain is provided in Figure 1. By acting on neurons and terminals expressing its receptor, NPS can alter the release of several neurotransmitters and thereby exert a direct or indirect modulation of the function of a very wide range of targets within the brain. For instance, NPS regulates amygdaloidal functions acting through different parallel pathways [7,8,44]. In the mouse, it enhances the glutamatergic tone to medial intercalated (mITC) GABAergic cells, presumably by acting on presynaptic NPSRs expressed in LA principal neurons. This phenomenon consequently increases the feedforward inhibition onto neurons in the CeA, which represents the main output nucleus of the amygdaloid complex [7]. In addition, NPS increases the feedforward inhibition toward BLA principal neurons, through a putative mechanism of action involving a direct excitation of principal neurons located in the endopiriform nucleus (EPN) [8]. A substantial difference has been found in the rat brain, as in control conditions, NPS failed to produce any significant effect in the monosynaptic glutamatergic release and feedforward GABAergic inhibition evoked into the CeA by the electrical stimulation of the BLA and entorhinal cortex, respectively [45]. Intriguingly, NPS was effective in an arthritis pain model, where it increased the mITC-mediated feedforward inhibition and decreased the release of glutamate into the CeA, indicating that specific conditions, such as the development of neuropathic pain, can produce plastic changes in the NPS–NPSR system [45]. Recent findings support the notion that NPS can also modulate neurotransmitter release in other brain regions. In the ventral hippocampus, NPS decreased basal glutamatergic neurotransmission and impaired long-term potentiation at the level of the CA3-CA1 synapses [46,47]. Furthermore, a recent study demonstrated that NPS indirectly inhibits the sleep-promoting galanin-expressing neurons in the VLPO by enhancing their GABAergic inputs, presumably through a direct depolarization of local galanin-negative GABAergic neurons [30]. The central administration of NPS increased cFos expression in the tuberomammillary nucleus wakefulness-promoting histaminergic neurons [21]. Direct and indirect evidence indicates that NPS interacts with monoaminergic signaling. NPSR is expressed in the ventral tegmental area (VTA) and the substantia nigra pars compacta (SNC) [2,41,43,48], suggesting that they could play a role in regulating the activity of the mesocorticolimbic dopamine (DA) pathway. Accordingly, neurochemical studies demonstrated that intra-VTA NPS microinfusion stimulates DA release in the nucleus accumbens (NAc) [49]. Similarly, the central injection of NPS increased the accumulation of DA and its metabolite 3,4-Dihydroxyphenylacetic acid in the medial prefrontal cortex (mPFC) in vivo [50]. However, only small amounts of the evoked DA release were detected ex vivo in cortical synaptosomes [51], indicating that synaptic terminals are presumably not the site of action for the NPS-dependent regulation of cortical DA release. Other information on the relationship between NPS and DA function arose from studies demonstrating that NPS stimulates the activity of SNC neurons, as suggested by an increased cFos immunoreactivity following NPS treatment, and enhanced release of local DA in the SNC following central NPS administration [48,52]. Concordantly, NPS successfully reversed the Parkinsonian-like motor deficits produced by the catecholaminergic neurotoxin 6-hydroxydopamine (6-OHDA) in mice and rats, and counteracted the decreased local DA release in the SNC produced by 6-OHDA treatment [48,53]. Although there is substantial agreement on the stimulatory properties of NPS on DAergic function, conflicting results have been reported regarding its role in the modulation of reward-related phenomena. Indeed, while several investigations observed no effects of NPS in producing conditioned place preference (CPP) or aversion [54,55,56], other work found a bidirectional effect of the peptide, where a lower dose (0.1 nmol) of NPS produced aversion, while a higher dose (1 nmol) exhibited rewarding-like properties [57]. This latter study also reported that rats moderately self-administer NPS intracranially in a cue-assisted operant paradigm [57]. However, given the known pro-cognitive and pro-attentive properties of NPS [3,58], this set of data could be interpreted as facilitation sign tracking induced by NPS [56,57] (see below for a more comprehensive discussion). Few studies analyzed the effects of NPS on regulating serotonin (5-hydroxytryptamine, 5-HT) release. NPS perfusion inhibited the evoked release of 5-HT in cortical and amygdaloidal synaptosome preparations [51,59]. Conversely, Si and colleagues [50] found that the central injection of NPS did not change mPFC concentrations of 5-HT and its metabolite 5-HIAA detected by in vivo microdialysis in rats. These discrepancies could depend on marked methodological differences given the moderate expression of NPSR in the 5-HTergic Raphe nucleus [2], which are not preserved in synaptosome preparations. Additional studies are needed to further establish how NPS regulates the 5-HTergic system and the potential functional readouts of these modulations. A number of reports, analyzing the action of NPS in memory formation and consolidation, have identified a functional interplay between NPS and the NAergic system. Recently, Okamura and colleagues [60] demonstrated that pretreatment with the beta-adrenergic receptor antagonist propranolol was effective in blocking the NPS-dependent enhancement of inhibitory avoidance memory consolidation. Similarly, propranolol abolished the pro-mnemonic effects of NPS on the novel object recognition test both when administered intracerebroventricularly (i.c.v.) or into the BLA [61], suggesting that the NPS-dependent memory enhancement could partially depend on an increased amygdaloidal NAergic tone. However, it is important to emphasize that NPSR is not expressed in the LC, the main brain source of NA, indicating that NPS does not directly excite LC NAergic cells. Therefore, the NPS-dependent modulation of NAergic function likely depends on NPSRs located in NAergic synaptic terminals or in other NAergic neuronal sources. Alternatively, NPS could interact with the NAergic system indirectly by modulating the activity of NPSR-expressing brain regions that, in turn, project to the LC. Supporting the notion that the NPSR expressed in NAergic terminals could play a role in the effects of the peptide, NPS inhibited the evoked release of NA in ex vivo cortical synaptosomes [51]. Finally, central infusions of NPS significantly enhanced the plasma concentration of adrenaline [62], indicating that adrenergic receptors located outside the brain could contribute to some extent to the effects of the peptide. Noteworthy, the biological activity of NPS is believed to be partially mediated by its interaction with other neuropeptidergic systems. NPSRs are expressed in Hcrt-1/Ox-A neurons [18,20,21], and NPS-positive axons are localized in the proximity of these neurons [18]. Accordingly, central NPS administration enhanced cFos expression in Hcrt-1/Ox-A-positive cells in the LH, in the DMH and in the PeF [18,20,21,63]. Additional indirect information on the interplay between NPS and the Hcrt-1/Ox-A system arises from behavioral experiments demonstrating that the self-administration of NPS is abolished in the presence of the Hcrt-1/Ox-A receptor (Ox1) selective antagonist SB-334867 [57]. Similarly, SB-334867 counteracted the exacerbation of cue-induced restatement of alcohol and cocaine seeking in rats produced by intra-LH microinfusions of NPS [19,20], an effect mediated by Hcrt-1/Ox-A receptors localized in the PVN and the bed nucleus of the stria terminalis (BNST), but not by those located in the LC and VTA [18]. On the other hand, a dense network of Hcrt-1/Ox-A fibers has been found near to NPS-expressing neurons in the periLC and, to a lesser extent, in the KF nucleus in mice. Furthermore, these data are corroborated by the fact that NPS-positive neurons in the periLC express Hcrt-2/Ox-B receptors [1]. Although additional work is needed to better establish, from a functional perspective, the existence of an orexinergic modulation of NPS release, altogether these findings suggest that the interaction between NPS and hypocretin/orexin systems could be of a bidirectional nature. NPS promotes the activation of the hypothalamic–pituitary–adrenal (HPA) axis, as indicated by the increased plasma concentrations of ACTH and corticosterone following intra-PVN NPS administration. This effect is believed to be indirect, since NPS did not promote ACTH release from anterior pituitary segments, but it did stimulate the release of CRF and vasopressin (but not neuropeptide Y) in hypothalamic explants [17]. Thus, it has been proposed that the activation of the HPA axis by NPS is mediated by the release of CRF and/or vasopressin from the PVN [17]. Intriguingly, it has been shown that the interaction between CRF and NPS systems is bidirectional. Indeed, CRF-positive fibers are localized in close proximity to periLC NPS-expressing neurons, and CRF perfusion directly depolarizes and increases the neuronal activity of periLC NPSergic cells in a CRF1 receptor-dependent manner [23]. Finally, a functional link between NPS and the OXT system within the PVN has also been reported. In fact, the NPSR is expressed in PVN neurons expressing OXT, and NPS perfusion activated OXT neurons in brain slices and induced the local release of OXT in vivo [32]. 

**Figure 2:**
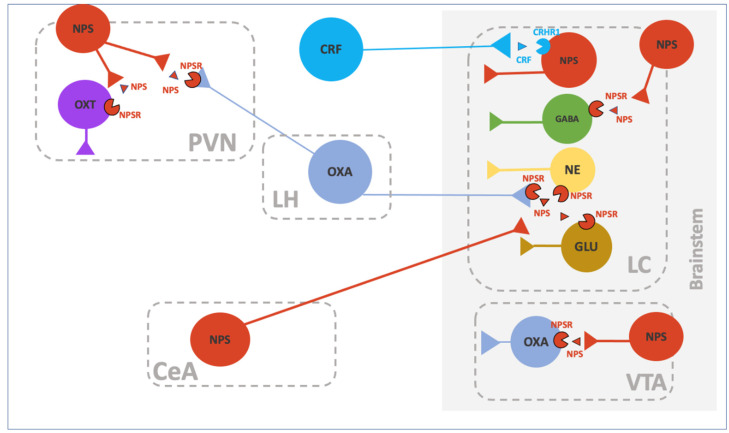
Schematic representation of the interaction of the NPS/NPSR system with other neurotransmitter systems. Ventral tegmental area (VTA); hypothalamic para ventricular nucleus (PVN): central amygdala (CeA); lateral hypothalamus (LH); locus coeruleus (LC).

The neurobiological interactions of NPS are summarized in Table 1. A schematic representation of the relationship of NPS/NPSR with other neurotransmitter systems is reported in Figure 2.

## 3. Role of NPS in AUD

AUD is a chronic relapsing disorder often associated with anxiety and maladaptive impulsivity [64,65,66]. The pro-arousal/anxiolytic profile of NPS suggested a potential role of this neuropeptide in the neurobiology of AUD [3]. Indeed, both variants of the NPSR gene have been associated with vulnerability to develop AUD [9]. In addition, compelling evidence of the involvement of the NPS/NPSR system in alcohol-related behavior has been provided by preclinical studies. 

### 3.1. NPS on Alcohol Drinking and Operant Self-Administration

Preclinical experiments in which the exogenous administration of NPS was tested on alcohol self-administration in the rat consistently reported a selective effect on rat lines genetically selected for their high alcohol preference. NPS reduced alcohol intake in a two-bottle choice (TBC) paradigm in Indiana alcohol-preferring (P) rats and operant self-administration on Marchigian-Sardinian alcohol-preferring (msP) rats, while it was not effective in non-preferring control lines [19,67,68]. A possible interpretation of this effect could be that the observed reduction in alcohol consumption was due to the NPS anorectic effects [15,69,70]. Indeed, alcohol represents an important source of calories for P and msP rats [71]. However, the reduction in alcohol self-administration was selective for alcohol-preferring lines [19,67,68] at doses that failed to reduce food intake [67]. In addition, the anorectic effect of NPS was observed in rat lines not selected for high alcohol preference [15,69,70]. Therefore, if this was the case, a reduction of self-administration should have been observed in non-preferring lines as well. A more compelling interpretation, supported by experimental evidence, is that the reduction in alcohol self-administration observed in alcohol-preferring rats [19,67,68] could be derived from the well-known anxiolytic properties of NPS [3,4,7,33,46,72,73,74,75]. Both msP and P rats express an innate anxious phenotype, and the anxiolytic effect of alcohol is a major driving force to consumption in these rats [71,76,77,78]. It is therefore likely that NPS decreased alcohol self-administration selectively in preferring lines by reducing the reinforcing value of the drug via its anxiolytic properties. In line with this interpretation, Enquist and colleagues demonstrated that NPS reduces alcohol consumption in a mouse TBC test, and that NPS anxiolytic and anti-depressive properties were enhanced in alcohol-exposed mice [55]. Psychological traits of withdrawal syndrome are key players in the development of AUD, as they are correlated with relapse risk and compulsive consumption [79,80]. Alcohol-intoxicated Wistar rats, expressing both physical and psychological withdrawal-like syndrome, showed increased NPSR gene expression in several nuclei of the amygdala and hypothalamic areas compared to non-intoxicated controls [81]. NPS alleviated withdrawal-induced anxiety in a defensive burying test [81]. This suggests that increased expression of NPS in stress-related areas could be an adaptive response to counteract anxiety symptoms associated with withdrawal. Interestingly, P and msP rats constitutively express psychological aspects typical of withdrawal syndrome, such as depression, anxiety, and heightened stress vulnerability [71,76,77]. Innate neurobiological and pharmacological response traits characteristic of a post-dependent state were reported in msP [82,83,84,85]. In line with post-dependent Wistars, the effect of NPS on alcohol consumption in P and msP rats was associated with the anxiolytic properties of this neuropeptide [19,67,68]. Furthermore, mice exposed to chronic alcohol drinking revealed increased anxiety and depression that was reverted by NPS pretreatment. The anxiolytic/antidepressant effects of NPS in these mice were mediated by the BLA, where NPS increased the amplitude of evoked GABA-mediated IPSCs [55]. It is also interesting to note that rats selectively bred for high anxiety (HAB) have a higher NPSR activity [86] akin to the human Ile107 risk variant [9,37] and that exogenous NPS treatment showed an anxiolytic effect in HAB rats, but not in their low-anxiety breeding counterpart [86]. Altogether, these data support the view that NPS reduced alcohol self-administration through its anxiolytic properties. In addition, data suggest the intriguing hypothesis that msP and P rats might have an increased NPSR expression and/or activity, making them more responsive to the anxiolytic effects of NPS, which would explain why NPS reduced alcohol self-administration in preferring rats, but not in non-preferring rats [19,67,68]. Though highly speculative at the present time, it is possible that the gain of function associated with the Ile107 NPSR variant, within specific brain areas, could co-segregate with traits predisposing to high anxiety and alcoholism vulnerability and it might represent a trait protecting against these predispositions. Somehow in contrast with the lack of effect of NPS on alcohol self-administration in Wistar rats [19], the inhibition of NPSR transmission by the antagonist NCGC00185684 decreased alcohol self-administration in this line [87]. Upon NPS stimulation, NPSR exerts its action by ERK phosphorylation, intra-cellular Ca2+ mobilization and increased cAMP levels, with a three-to-four times higher potency on the ERK pathway [9,87]. NCGC00185684 blocked in vitro NPS-induced ERK phosphorylation studies and in vivo alcohol-induced ERK phosphorylation in the CeA and in the shell region of NAc, therefore this was proposed as the mechanism of action by which NCGC00185684 decreased alcohol self-administration [87]. This encourages a possible interpretation for the lack of an exogenous NPS effect on alcohol self-administration in Wistars. Indeed, the ceiling ERK phosphorylation induced by alcohol might have covered NPS effects, leaving self-administration level unaltered, whereas NCGC00185684, by preventing alcohol-induced ERK phosphorylation, decreased alcohol self-administration. As to why NPS agonism decreased self-administration selectively in preferring lines [19,67,68], whereas NPS antagonism decreased self-administration in heterogeneous Wistar rats [87], remains unclear. The CeA has been proposed as a site of action of NCGC00185684 in non-dependent Wistar rats [87], whereas neurobiological adaptations of the NPS/NPSR system observed in post-dependent Wistar rats included increased expression of NPSR in the BLA, PaV and LH, but not in the CeA [81]. In addition, the anxiolytic properties of NPS mediating the reduction of alcohol consumption in mice have been demonstrated to ground on the BLA [55]. Therefore, it would be worth testing how alcohol-preferring lines respond to NCGC00185684, and test whether NCGC00185684 in Wistar and NPS in msP and P rats act through different neurocircuitries.

### 3.2. NPS on Reinstatement of Alcohol Seeking

Exposure to the environmental stimuli associated with alcohol experience and their interaction with stressful events is recognized as a major factor augmenting relapse risks [88,89]. We demonstrated that in outbred Wistar rats, NPS can prime the reinstatement of alcohol seeking [68] and exert a facilitatory role on cued reinstatement through interrogation of the hypothalamic Hcrt-1/Ox-A system [18,19]. The downstream activation of Hcrt-1/Ox-A clearly indicates that the relapse facilitatory action of NPS is mediated by the stress/pro-arousal component of this neuropeptide’s psychopharmacology [3]. Because Hcrt-1/Ox-A, the downstream modulator of NPS facilitatory role on relapse [18,19], was reported to mediate alcohol self-administration as well [90,91,92], the co-existence of NPS’s facilitatory action on relapse with the lack of effect on self-administration in Wistar may appear controversial. However, while the site of action by which hypocretin/orexin modulates alcohol self-administration is the VTA [93], this area plays no role in the Hcrt-1/Ox-A-mediated facilitation of relapse induced by NPS [18]. In fact, in a series of histological and pharmacological studies, we explored the neurocircuitry by which NPS facilitates cued reinstatement. We demonstrated that site-specific pretreatment with the selective OX1 receptor antagonist SB334867 blocked NPS-induced facilitation of relapse-like behavior when SB334867 was delivered within the BNST and PVN, but not when the pretreatment was given in the VTA and LC [18]. Our findings were further corroborated by histological analyses demonstrating that: (i) NPS fibers run in close opposition to Hcrt-1/Ox-A neurons in the LH [18]; (ii) LH Hcrt-1/Ox-A neurons express NPSR [18]; (iii) NPS induces cFos activation in hypothalamic Hcrt-1/Ox-A neurons [20]; (iv) retro-tracing marker injected within the PVN and BNST (i.e., the sites where SB334867 blocked NPS) co-labels with LH Hcrt-1/Ox-A neurons [18]. Altogether, our findings demonstrate that NPS facilitates relapse-like behavior through LH Hcrt-1/Ox-A neurons, which, in turn, interrogate the extended amygdala via BNST and the HPA axis via PVN (see also [17]). In summary, alcohol self-administration and relapse studies demonstrated that, consistently with its dual pro-arousal/anxiolytic profile, NPS exerts a double action on alcohol seeking behavior. The stress-related component of NPS psychopharmacology promotes relapse via the interrogation of LH Hcrt-1/Ox-A neurons and, in turn, by Hcrt-1/Ox-A-responsive BNST and PVN neurons. On the contrary, the action on alcohol self-administration seems to depend on the anxiolytic action of NPS that could be mediated by the BLA.

## 4. NPS and Reward

As discussed above, NPS did not affect drug self-administration in outbred rodent lines when self-administration was maintained by a positive reinforcement mechanism. Indeed, NPS decreased alcohol self-administration in P rats [67], msP rats [68], and mice seeking alcohol to alleviate their anxious state [55]. However, when tested on outbred lines in a non-dependent state, NPS failed to affect cocaine [20], alcohol [19,67] and nicotine (Cannella et al., unpublished observation) self-administration. It might still be interesting to assess whether NPS plays a role when excessive drinking and alcohol reinforcement is mediated by nicotine administration [94,95]. We and others reported that NPS induced neither place preference nor aversion, in a place conditioning paradigm [54,55,56]. Moreover, Li and colleagues reported that NPS blocked the acquisition of morphine CPP [54]. Altogether, these data indicate that NPS is devoid of rewarding properties per se. However, in favor of a possible rewarding effect of NPS, it was reported that NPS can increase DA release in the mPFC and NAc [49,50]. In addition, Cao and coworkers demonstrated that rats can actively self-administer NPS, and NPS self-administration was reduced by the selective D1-like receptor antagonist SCH 23390, and by the selective OX1 antagonist SB-334867 [57]. In the same work, and in contrast with others [54,55,56], it was reported that 1 nmol of NPS induced CPP and 0.1 nmol induced aversion [57]. Thus, the work from Cao et al. would indicate a rewarding effect of NPS in contrast with the rest of the literature. However, an alternative explanation could be proposed to reconcile this apparently contrasting result on NPS’s reinforcing effects. In the work of Cao and colleagues, intraventricular self-infusions were paired with discrete cue light. Notably, this experiment included a control group that self-administered saline in the same condition as the NPS-treated group (self-administered about 20 infusions of saline/session), suggesting that rats actively pressed to visualize the discrete cue (unfortunately an inactive lever control was not included in the experimental design). Though significantly higher, the NPS-reinforced groups showed a level of self-administration only 0.5 times higher than the saline control group [57]. Therefore, an alternative interpretation could be that the observed NPS self-infusion behavior was secondary to NPS’s ability to facilitate sign tracking. This interpretation would be consistent with the pro-cognitive and vigilance-enhancing properties of NPS [7,8]. 

## 5. Role of NPS in Cocaine Seeking

As for alcohol-related behaviors, the role of the NPS system in the regulation of cocaine properties is under extensive scrutiny. Since the publication of the first paper in 2009 [22], more publications exploring the effects of either activation or inhibition of NPS receptors on cocaine-related behaviors have been published. Here, we report an up-to-date overview of the current literature on the role of the NPS system in modulating cocaine intake and the reinstatement of cocaine seeking.

### 5.1. Role of the NPS System in Cocaine-Induced Reward

To date, few studies have explored the effects of exogenous NPS on cocaine self-administration. Our laboratory reported that i.c.v. infusion of NPS (1.0, and 2.0 nmol) failed to reduce cocaine self-administration [20]. Moreover, in the same study, we show that intraperitoneal injection (i.p.) of the selective NPS receptor antagonist SHA 68 (30.0, and 60 mg/kg) [96] did not modify cocaine intake under the same schedule of reinforcement [20]. In another set of experiments [97], we obtained similar results using another selective NPS receptor antagonist NPSR-QA1 [98]. This compound (15 and 30 mg/kg, i.p.) blunted food self-administration in rats, without affecting cocaine intake [97]. However, it was recently shown that the NPSR antagonist RTI-118 [99] was able to reduce cocaine and food self-administration in rats [100]. Interestingly, RTI-118 was able to selectively reduce cocaine self-administration at the lower doses (10–20 mg/kg, i.p.), without affecting food self-administration, suggesting a selective effect for cocaine at this range of doses. The higher solubility of RTI-118 in water at physiological pH compared to SHA 68 could lead to better bioavailability, thereby explaining the discrepancies between the two studies [96]. More recently, another study found that RTI-118 (3.0, 10.0 and 32.0 mg/kg, i.p.) produced a dose-dependent blockade of the cocaine-induced facilitation of intracranial self-stimulation (ICSS) in rats at a range of doses that induced little or no effect on ICSS when administered alone [101]. Further studies are needed to better clarify the different effects of NPSR antagonists on cocaine self-administration and to elucidate the role of the NPS/NPSR system in cocaine reinforcement.

### 5.2. Role of the NPS System in the Reinstatement of Cocaine Seeking

More straightforward is the effect of NPS system in the regulation of cocaine seeking and relapse [20,22,96,97]. In 2009, Paneda and colleagues demonstrated that i.c.v. administration of NPS (0.45 nmol) was able to facilitate the reinstatement of cocaine-seeking behavior in mice [22]. This effect was dependent on the manipulation of the CRF system, as it was prevented by pretreatment with the CRF1 receptor antagonist antalarmin (30 mg/kg, i.p.) [22]. Accordingly, in CRF receptor knockout ((CRF1 (-/-)) mice, NPS (0.45 nmol) failed to reinstate extinguished lever pressing for cocaine and to stimulate locomotor activity [22]. These results indicate that the NPS-induced reinstatement of cocaine-seeking was mediated by stress-like effects. Over the following years, these results were replicated in rats [20]. I.c.v. and intra-VTA administration of NPS facilitated the reinstatement of cocaine-induced CPP in mice [101], corroborating the initial findings on cocaine-seeking behavior [20,22]. In addition, the NPSR antagonist SHA 68 (50 mg/kg, i.p.) blocked the stress-induced reinstatement of extinguished cocaine CPP [101]. In 2011, our laboratory demonstrated that i.c.v. or intra-LH infusions of NPS (1.0 and 2.0 nmol) promoted cocaine-seeking behavior in a discriminative cue-induced reinstatement model, whereas a smaller, but significant effect was detected when the peptide was delivered into the PeF, but not into the DMH or the CeA. Accordingly, the administration of SHA 68 (30.0 and 60.0 mg/kg) decreased lever pressing induced by environmental stimuli previously associated with cocaine availability [20]. Similarly, in another study, we reported that the other two NPS receptor antagonists, NPSR-QA1 (15.0 and 30.0 mg/kg, i.p.) and (D-Cys(tBut)5)NPS (10.0, 30.0 and 60.0 nmol) were able to reduce the cue-induced reinstatement of cocaine seeking, with a stronger effect for (D-Cys(tBut)5)NPS (10.0 and 30.0 nmol) when it was specifically microinjected in the PeF and the LH, but not in the CeA [97]. Likewise, Schmoutz and colleagues [96] reported that the NPS receptor antagonist RTI-118 (1.0, 5.0, 10.0 and 20.0 mg/kg, i.p.) decreased the primed-, yohimbine- and cued reinstatement of cocaine seeking. Overall, these studies indicate that NPS receptor antagonism may be a useful strategy to prevent relapse to cocaine, whereas the activation of NPS receptors through NPS infusion promotes cue-induced relapse to cocaine-seeking behavior. This latter effect can be explained by the general peptide’s ability to increase goal-oriented behaviors [3]. However, in a paradigm of discriminative cue-induced reinstatement (cocaine paired with a tone vs. saline paired with house light), we demonstrated that the i.c.v. administration of NPS did not affect lever responding for cues previously associated with saline delivery [20], thus strengthening the idea that the exacerbation of reinstatement induced by NPS was not secondary to its action on locomotor activity or arousal. Together with the CRF system, the hypothalamic Hcrt-1/Ox-A system is implicated in the regulation of NPS’s effect on cocaine relapse as well. Indeed, we reported that NPS increased cFos expression in the hypothalamic Hcrt1/Ox-A cells [19,20], and intra-LH injections of NPS (0.5 nmol) markedly increased the cue-induced reinstatement of cocaine seeking [20], an effect that was abolished by pretreatment with the selective Ox-1 receptor antagonist SB-334867 (10mg/kg, i.p.). In agreement with these data, more recently, Chou and collaborators [102] demonstrated that i.c.v. infusions of NPS (1 nmol) augmented cFos-containing orexin neurons in the LH and the Ox-A level in the VTA [102]. This latter effect was prevented by the NPS receptor antagonist SHA 68 (50 mg/kg, i.p.), suggesting that NPS activates an orexinergic neurocircuitry involving the hypothalamus and the VTA. Noteworthy, the NPS-induced reinstatement of cocaine CPP was suppressed by systemic (10 mg/kg, i.p.) and intra-VTA (15 nmol) injection of SB-334867, suggesting a crucial role of the orexinergic signaling in the VTA in mediating such effect [102]. Overall, these results indicated that NPS is released under stressful conditions and activates LH orexinergic neurons to facilitate orexin release in the VTA, subsequently leading to the reinstatement of cocaine CPP through Hcrt1/Ox-A receptor signaling. However, considering that it was demonstrated that restraint stress can activate both the Hcrt1/Ox-A and the CRF systems [103], and CRF signaling is involved in modulating NPS’s facilitation of cocaine-seeking [22], a primary contribution of the CRF system cannot be ruled out. The data described so far demonstrated that the NPS system modulates cocaine relapse through the activation of both the Hcrt1/Ox-A and the CRF systems [20,22,102,103], but the precise mechanisms of action are not clear yet. Several reports have shown that there may be direct interactions between CRF and Hcrt1/Ox-A systems, especially in the VTA [104]. Indeed, it was demonstrated that CRF-immunopositive cells are in direct contact with Hcrt1/Ox-A neurons in the LH, and that several Hcrt1/Ox-A cells expressed CRF receptors [105]. In addition, Sakamoto et al. (2004) reported that Hcrt-1/Ox-A activates approximately 96% and 45% of CRF-containing neurons in the PVN and the CeA, respectively [106]. This, in turn, increases CRF and vasopressin expression in the PVN and activates the HPA axis [107]. It is well established that both Hcrt1/Ox-A and CRF increase VTA DAergic neuron activity and potentiate NMDAR-mediated synaptic transmission in these cells [104,108,109,110]. When microinjected in the VTA, both neuropeptides promote DA release in the NAc and PFC [104,111,112,113], and they induce reinstatement to cocaine seeking in rats [111,114]. However, they may promote reinstatement to cocaine seeking by independent mechanisms. Indeed, it was shown that the effect of intra-VTA administration of Hcrt1/Ox-A can be abolished by Hcrt1/Ox-A antagonist SB-408124, but not by CRF receptor antagonism [114], and the Ox1 receptor antagonist SB-408124 did not block CRF-dependent foot-shock-induced reinstatement [114]. Moreover, the same work demonstrated that the reinstatement of cocaine seeking by intra-VTA infusion of CRF is completely glutamate-dependent, whereas reinstatement induced by intra-VTA Hcrt1/Ox-A infusion is not, suggesting a separate mechanism of action within this circuitry [114]. Taken together, these data indicate that these two peptidergic systems can work in parallel through distinct mechanisms and that NPS could modulate the two systems independently. A deeper understanding of these complex interactions would provide useful tools to find more effective therapies to treat cue- and stress-induced relapse in abstinent cocaine-dependent individuals.

A summary of the main preclinical findings reviewed above is provided in Table 2.

Altogether, the preclinical and genetic data indicate that NPS is likely to play a role in drug abuse. This makes the NPS a potential target to treat drug use disorders. Yet the panel of molecules developed to target NPSR is small, and to the best of our knowledge, none of them have entered clinical trials. However, it is interesting to observe that the orexin/hypoceretin system is a major downstream target by which NPS exacerbates the reinstatement of drug seeking, and a certain number of trials are testing orexin antagonists in patients diagnosed AUD, opioid and cocaine use disorders (Table 3).

## 6. Conclusive Remarks

Here, we reviewed the evidence that NPS is a modulator of catecholamines, GABA and glutamate activities. NPS also interacts with key players of both the peripheral and central stress response system—specifically with Hcrt-1/Ox-A and CRF. This wide spectrum of interactions is associated with a unique physio-pharmacological profile, as this neurotransmitter promotes arousal and is anxiolytic at the same time. The pharmacological traits and neurobiological interactions of NPS indicated this neuropeptide as a new player in the stress response neurosystem. The dual pharmacology of NPS as player of the stress system is reflected by its effect on drug self-administration and the reinstatement of drug seeking. On the one hand, NPS reduced alcohol self-administration in rodents consuming alcohol to self-medicate their innate or withdrawal-induced anxiety state, and this effect was associated with the anxiolytic effect of NPS. On the other hand, NPS primed extinguished alcohol and cocaine seeking, and this latter effect was demonstrated to be mediated by CRF. In addition, NPS exacerbated the cued reinstatement of alcohol and cocaine seeking through Hcrt-1/Ox-A. The emergence of the NPS as a new player of the stress system involved in addiction is noteworthy as, although stress plays a major and well-consolidated role in addiction, no drugs targeting the stress system to treat addiction have hit the market so far. We predict that NPSR agonists would be indicated to help quitting alcohol consumption and to mitigate the psychological aspects of alcohol withdrawal syndrome; interestingly, the first NPSR agonist has been developed recently [115] and it would be interesting to test it on alcohol self-administration. Conversely, NPSR antagonists would be indicated to prevent relapse. The therapeutic potential of targeting the NPS system is not limited to this, though. Indeed, despite the exogenous administration of NPS having no effect on alcohol and cocaine self-administration in non-preferring rats, NPSR antagonists reduced the self-administration of both drugs. Future studies should be directed to characterize the neurocircuitries through which NPS reduces alcohol self-administration in preferring rat lines and to understand the neurobiological bases of the efficacy of NPSR antagonists where the exogenous administration of the peptide was ineffective. 

## Figures and Tables

**Table 1:** Neurobiological effects of NPS.

Brain System	Animal	Sex	Route of Administration	Experimental Procedure	Effect	Ref.
DA system	Wistar rats	Male	Intra-VTA injection	In vivo microdyalisis	↑ DA release in the Nac	[55]
	Sprague Dawley rats	Male	Central injection (i.c.v.)	In vivo microdyalisis	↑ DA release in the mPFC	[56]
	Swiss mice	N/A	Bath perfusion	Ex vivo synaptosomes	Little ↑ effects on evoked DA release in cortical synaptosomes	[57]
	Wistar rats/Swiss mice	Male	Central injection (i.c.v.)	cFOS immunodetection	↑ cFOS expression in SNC DA neurons	[54,58]
	Wistar rats	Male	Central injection (i.c.v.)	In vivo microdyalisis	↑ DA local release in the SNC of 6-OHDA treated rats	[54]
5-HT system	Swiss mice	N/A	Bath perfusion	Ex vivo synaptosomes	↓ evoked 5-HT release in cortical and amygdaloidal synaptosomes	[57]
	Sprague Dawley rats	Male	Central injection (i.c.v.)	In vivo microdyalisis	No effects on 5-HT in the mPFC	[56]
NA system	Swiss mice	N/A	Bath perfusion	Ex vivo synaptosomes	Little effects on evoked DA release in cortical synaptosomes	[57]
Limbic system	C57BL/6J mice	N/A	Bath perfusion	Ex vivo patch-clamp recordings	↑ glutamate release on mITC neurons and ↑ feedforward inhibition on CeA neurons	[15]
	GAD67-GFP mice	N/A	Bath perfusion	Ex vivo patch-clamp recordings	↑ feedforward inhibition on BLA principal neurons and ↑ excitation of EPN principal neurons	[16]
	Sprague Dawley rats	Male	Bath perfusion	Ex vivo patch-clamp recordings	No effects on evoked glutamate release and feedforward inhibition the CeA of control animals	[51]
					↓ evoked glutamate release and ↑ feedforward inhibition in the CeA in a neuropathic pain model	
	C57BL/6N mice	Male	Bath perfusion/intranasal	Ex vivo field potential recordings	↓ Paired pulse ratio and impaired LTP in CA3-CA1 synapses of the ventral hippocampus	[52,53]
Hypothalamus and HPA axis	Long Evans, Sprague Dawley, Wistar rats	Male	Central injection (i.c.v.)	cFOS immunodetection	↑ cFOS expression in LH, DMH and PeF Hcrt-1/Ox-A neurons	[25,27,28]
	Sprague Dawley rats	Male	Central injection (i.c.v.)	cFOS immunodetection	↑ cFOS expression in TMN histaminergic neurons	[28]
	C57BL/6J mice	Male	Bath perfusion	Ex vivo patch-clamp recordings	↑ neuronal activity of non-galanin VLPO neurons	[37]
					↓ neuronal activity of galanin VLPO neurons by ↑ GABA release	
	Wistar rats	Male	Bath perfusion	Ex vivo GCamp6 calcium signaling	↑ OXT neuronal activity	[39]
			Central injection (i.c.v.)	In vivo microdyalisis	↑ OXT local release	
	Wistar rats	Male	Central injection (i.c.v.)	Plasma concentration	↑ concentration ACTH and corticosterone	[24]
			Bath perfusion	Hypothalamic explants	↑ CRF and vasopressin release from anterior pituitary segments, no effects on ACTH and NPY	

**Table 2:** Main preclinical findings on probing the NPS/NPSR system in alcohol and cocaine seeking.

Drug of Abuse	Strain	Drug	Route	Paradigm	Result	Ref.
Alcohol	P rats	NPS	i.c.v.	TBC	Reduced alcohol intake	[66]
Alcohol	Wistar rats	NPS	i.c.v. and LH	Cued reinstatement	Exacerbated seeking	[18]
Alcohol	Wistar rats	NPS	i.c.v.	Post-dependent alcohol withdrawal	Alleviated symptoms	[80]
Alcohol	Mice	NPS	i.c.v.	TBC	Reduced alcohol intake	[54]
Alcohol	Wistar rats	NPSR antagonist NCGC00185684	i.p.	Fixed ratio self-administration	Decreased self-administration	[86]
Alcohol	Wistar rats	NPS	LH	Cued reinstatement	Exacerbated seeking	[17]
Alcohol	Wistar and msP rats	NPS	i.c.v.	Reinstatement of seeking and self-administration	Reinstated seeking in Wistars and reduced self-administration in msP	[67]
Cocaine	Wild-type and CRF1 KO mice	NPS	i.c.v.	Reinstatement of seeking	Reinstated seeking in wild-type but not CRF1KO mice	[21]
Cocaine	Long Evans rats	NPS	i.c.v. and LH	Cued reinstatement	Exacerbated seeking	[19]
Cocaine	Wistar rats	NPSR antagonist RTI118	i.p.	Cued reinstatement of seeking and self-administration	Decreased both	[99]
Cocaine	Long Evans rats	NPSR antagonist NPSR-QA1	i.p.	Cued reinstatement of seeking	Reduced seeking	[96]

**Table 3:** Clinical trial on drug use disorders targeting the orexin system.

NCT Number	Disease	Treatment	Phase
NCT03897062	Alcohol Use Disorder	Suvorexant	2
NCT04229095	Alcohol Use Disorder	Suvorexant	2
NCT04287062	Opioid Use Disorder	Suvorexant	2
NCT04262193	Opioid Use Disorder	Suvorexant	2
NCT03789214	Opioid Use Disorder	Suvorexant	2
NCT05145764	Opioid Use Disorder	Suvorexant	2
NCT04818086	Opioid Use Disorder	Lemborexant	1 and 2
NCT02785406	Cocaine Use Disorder	Suvorexant	2
NCT03937986	Cocaine Use Disorder	Suvorexant	1

## Data Availability

Data sharing not applicable.

## References

[1] Liu X., Zeng J., Zhou A., Theodorsson E., Fahrenkrug J., Reinscheid R.K. (2011). Molecular fingerprint of neuropeptide S-producing neurons in the mouse brain. J. Comp. Neurol..

[2] Xu Y.L., Gall C.M., Jackson V.R., Civelli O., Reinscheid R.K. (2007). Distribution of neuropeptide S receptor mRNA and neurochemical characteristics of neuropeptide S-expressing neurons in the rat brain. J. Comp. Neurol..

[3] Xu Y.L., Reinscheid R.K., Huitron-Resendiz S., Clark S.D., Wang Z., Lin S.H., Brucher F.A., Zeng J., Ly N.K., Henriksen S.J. (2004). Neuropeptide S: A neuropeptide promoting arousal and anxiolytic-like effects. Neuron.

[4] Rizzi A., Vergura R., Marzola G., Ruzza C., Guerrini R., Salvadori S., Regoli D., Calo G. (2008). Neuropeptide S is a stimulatory anxiolytic agent: A behavioural study in mice. Br. J. Pharmacol..

[5] Donner J., Haapakoski R., Ezer S., Melen E., Pirkola S., Gratacos M., Zucchelli M., Anedda F., Johansson L.E., Soderhall C. (2010). Assessment of the neuropeptide S system in anxiety disorders. Biol. Psychiatry.

[6] Zoicas I., Menon R., Neumann I.D. (2016). Neuropeptide S reduces fear and avoidance of con-specifics induced by social fear conditioning and social defeat, respectively. Neuropharmacology.

[7] Jungling K., Seidenbecher T., Sosulina L., Lesting J., Sangha S., Clark S.D., Okamura N., Duangdao D.M., Xu Y.L., Reinscheid R.K. (2008). Neuropeptide S-mediated control of fear expression and extinction: Role of intercalated GABAergic neurons in the amygdala. Neuron.

[8] Meis S., Bergado-Acosta J.R., Yanagawa Y., Obata K., Stork O., Munsch T. (2008). Identification of a neuropeptide S responsive circuitry shaping amygdala activity via the endopiriform nucleus. PLoS ONE.

[9] Reinscheid R.K., Xu Y.L., Okamura N., Zeng J., Chung S., Pai R., Wang Z., Civelli O. (2005). Pharmacological characterization of human and murine neuropeptide s receptor variants. J. Pharmacol. Exp. Ther..

[10] Raczka K.A., Gartmann N., Mechias M.L., Reif A., Buchel C., Deckert J., Kalisch R. (2010). A neuropeptide S receptor variant associated with overinterpretation of fear reactions: A potential neurogenetic basis for catastrophizing. Mol. Psychiatry.

[11] Okamura N., Hashimoto K., Iyo M., Shimizu E., Dempfle A., Friedel S., Reinscheid R.K. (2007). Gender-specific association of a functional coding polymorphism in the Neuropeptide S receptor gene with panic disorder but not with schizophrenia or attention-deficit/hyperactivity disorder. Prog. Neuropsychopharmacol. Biol. Psychiatry.

[12] Dannlowski U., Kugel H., Franke F., Stuhrmann A., Hohoff C., Zwanzger P., Lenzen T., Grotegerd D., Suslow T., Arolt V. (2011). Neuropeptide-S (NPS) receptor genotype modulates basolateral amygdala responsiveness to aversive stimuli. Neuropsychopharmacology.

[13] Gechter J., Liebscher C., Geiger M.J., Wittmann A., Schlagenhauf F., Lueken U., Wittchen H.U., Pfleiderer B., Arolt V., Kircher T. (2019). Association of NPSR1 gene variation and neural activity in patients with panic disorder and agoraphobia and healthy controls. Neuroimage Clin..

[14] Domschke K., Reif A., Weber H., Richter J., Hohoff C., Ohrmann P., Pedersen A., Bauer J., Suslow T., Kugel H. (2011). Neuropeptide S receptor gene—converging evidence for a role in panic disorder. Mol. Psychiatry.

[15] Fedeli A., Braconi S., Economidou D., Cannella N., Kallupi M., Guerrini R., Calo G., Cifani C., Massi M., Ciccocioppo R. (2009). The paraventricular nucleus of the hypothalamus is a neuroanatomical substrate for the inhibition of palatable food intake by neuropeptide S. Eur. J. Neurosci..

[16] Botticelli L., Micioni Di Bonaventura E., Ubaldi M., Ciccocioppo R., Cifani C., Micioni Di Bonaventura M.V. (2021). The Neural Network of Neuropeptide S (NPS): Implications in Food Intake and Gastrointestinal Functions. Pharmaceuticals.

[17] Smith K.L., Patterson M., Dhillo W.S., Patel S.R., Semjonous N.M., Gardiner J.V., Ghatei M.A., Bloom S.R. (2006). Neuropeptide S stimulates the hypothalamo-pituitary-adrenal axis and inhibits food intake. Endocrinology.

[18] Ubaldi M., Giordano A., Severi I., Li H., Kallupi M., de Guglielmo G., Ruggeri B., Stopponi S., Ciccocioppo R., Cannella N. (2016). Activation of Hypocretin-1/Orexin-A Neurons Projecting to the Bed Nucleus of the Stria Terminalis and Paraventricular Nucleus Is Critical for Reinstatement of Alcohol Seeking by Neuropeptide S. Biol. Psychiatry.

[19] Cannella N., Economidou D., Kallupi M., Stopponi S., Heilig M., Massi M., Ciccocioppo R. (2009). Persistent increase of alcohol-seeking evoked by neuropeptide S: An effect mediated by the hypothalamic hypocretin system. Neuropsychopharmacology.

[20] Kallupi M., Cannella N., Economidou D., Ubaldi M., Ruggeri B., Weiss F., Massi M., Marugan J., Heilig M., Bonnavion P. (2010). Neuropeptide S facilitates cue-induced relapse to cocaine seeking through activation of the hypothalamic hypocretin system. Proc. Natl. Acad. Sci. USA.

[21] Zhao P., Shao Y.F., Zhang M., Fan K., Kong X.P., Wang R., Hou Y.P. (2012). Neuropeptide S promotes wakefulness through activation of the posterior hypothalamic histaminergic and orexinergic neurons. Neuroscience.

[22] Paneda C., Huitron-Resendiz S., Frago L.M., Chowen J.A., Picetti R., de Lecea L., Roberts A.J. (2009). Neuropeptide S reinstates cocaine-seeking behavior and increases locomotor activity through corticotropin-releasing factor receptor 1 in mice. J. Neurosci..

[23] Jungling K., Liu X., Lesting J., Coulon P., Sosulina L., Reinscheid R.K., Pape H.C. (2012). Activation of neuropeptide S-expressing neurons in the locus coeruleus by corticotropin-releasing factor. J. Physiol..

[24] Lee M.T., Chiu Y.T., Chiu Y.C., Hor C.C., Lee H.J., Guerrini R., Calo G., Chiou L.C. (2020). Neuropeptide S-initiated sequential cascade mediated by OX1, NK1, mGlu5 and CB1 receptors: A pivotal role in stress-induced analgesia. J. Biomed. Sci..

[25] Jungling K., Lange M.D., Szkudlarek H.J., Lesting J., Erdmann F.S., Doengi M., Kugler S., Pape H.C. (2015). Increased GABAergic Efficacy of Central Amygdala Projections to Neuropeptide S Neurons in the Brainstem During Fear Memory Retrieval. Neuropsychopharmacology.

[26] Domi E., Xu L., Toivainen S., Nordeman A., Gobbo F., Venniro M., Shaham Y., Messing R.O., Visser E., van den Oever M.C. (2021). A neural substrate of compulsive alcohol use. Sci. Adv..

[27] Haubensak W., Kunwar P.S., Cai H., Ciocchi S., Wall N.R., Ponnusamy R., Biag J., Dong H.W., Deisseroth K., Callaway E.M. (2010). Genetic dissection of an amygdala microcircuit that gates conditioned fear. Nature.

[28] Zhang S., Jin X., You Z., Wang S., Lim G., Yang J., McCabe M., Li N., Marota J., Chen L. (2014). Persistent nociception induces anxiety-like behavior in rodents: Role of endogenous neuropeptide S. Pain.

[29] Jiang J.H., Peng Y.L., Zhang P.J., Xue H.X., He Z., Liang X.Y., Chang M. (2018). The ventromedial hypothalamic nucleus plays an important role in anxiolytic-like effect of neuropeptide S. Neuropeptides.

[30] Chauveau F., Claverie D., Lardant E., Varin C., Hardy E., Walter A., Canini F., Rouach N., Rancillac A. (2020). Neuropeptide S promotes wakefulness through the inhibition of sleep-promoting ventrolateral preoptic nucleus neurons. Sleep.

[31] Barchiesi R., Chanthongdee K., Domi E., Gobbo F., Coppola A., Asratian A., Toivainen S., Holm L., Augier G., Xu L. (2021). Stress-induced escalation of alcohol self-administration, anxiety-like behavior, and elevated amygdala Avp expression in a susceptible subpopulation of rats. Addict. Biol..

[32] Grund T., Goyon S., Li Y., Eliava M., Liu H., Charlet A., Grinevich V., Neumann I.D. (2017). Neuropeptide S Activates Paraventricular Oxytocin Neurons to Induce Anxiolysis. J. Neurosci..

[33] Leonard S.K., Dwyer J.M., Sukoff Rizzo S.J., Platt B., Logue S.F., Neal S.J., Malberg J.E., Beyer C.E., Schechter L.E., Rosenzweig-Lipson S. (2008). Pharmacology of neuropeptide S in mice: Therapeutic relevance to anxiety disorders. Psychopharmacology.

[34] Han R.W., Yin X.Q., Chang M., Peng Y.L., Li W., Wang R. (2009). Neuropeptide S facilitates spatial memory and mitigates spatial memory impairment induced by N-methyl-D-aspartate receptor antagonist in mice. Neurosci. Lett..

[35] Liu X., Si W., Garau C., Jungling K., Pape H.C., Schulz S., Reinscheid R.K. (2017). Neuropeptide S precursor knockout mice display memory and arousal deficits. Eur. J. Neurosci..

[36] Reinscheid R.K. (2007). Phylogenetic appearance of neuropeptide S precursor proteins in tetrapods. Peptides.

[37] Laas K., Reif A., Akkermann K., Kiive E., Domschke K., Lesch K.P., Veidebaum T., Harro J. (2015). Neuropeptide S receptor gene variant and environment: Contribution to alcohol use disorders and alcohol consumption. Addict. Biol..

[38] Laas K., Reif A., Kiive E., Domschke K., Lesch K.P., Veidebaum T., Harro J. (2014). A functional NPSR1 gene variant and environment shape personality and impulsive action: A longitudinal study. J. Psychopharmacol..

[39] Fontenelle L.F., Oostermeijer S., Harrison B.J., Pantelis C., Yucel M. (2011). Obsessive-compulsive disorder, impulse control disorders and drug addiction: Common features and potential treatments. Drugs.

[40] Laas K., Reif A., Akkermann K., Kiive E., Domschke K., Lesch K.P., Veidebaum T., Harro J. (2014). Interaction of the neuropeptide S receptor gene Asn(1)(0)(7)Ile variant and environment: Contribution to affective and anxiety disorders, and suicidal behaviour. Int. J. Neuropsychopharmacol. Off. Sci. J. Coll. Int. Neuropsychopharmacol..

[41] Clark S.D., Duangdao D.M., Schulz S., Zhang L., Liu X., Xu Y.L., Reinscheid R.K. (2011). Anatomical characterization of the neuropeptide S system in the mouse brain by in situ hybridization and immunohistochemistry. J. Comp. Neurol..

[42] Adori C., Barde S., Bogdanovic N., Uhlen M., Reinscheid R.R., Kovacs G.G., Hokfelt T. (2015). Neuropeptide S- and Neuropeptide S receptor-expressing neuron populations in the human pons. Front. Neuroanat..

[43] Leonard S.K., Ring R.H. (2011). Immunohistochemical localization of the neuropeptide S receptor in the rat central nervous system. Neuroscience.

[44] Meis S., Stork O., Munsch T. (2011). Neuropeptide S-mediated facilitation of synaptic transmission enforces subthreshold theta oscillations within the lateral amygdala. PLoS ONE.

[45] Ren W., Kiritoshi T., Gregoire S., Ji G., Guerrini R., Calo G., Neugebauer V. (2013). Neuropeptide S: A novel regulator of pain-related amygdala plasticity and behaviors. J. Neurophysiol..

[46] Ionescu I.A., Dine J., Yen Y.C., Buell D.R., Herrmann L., Holsboer F., Eder M., Landgraf R., Schmidt U. (2012). Intranasally administered neuropeptide S (NPS) exerts anxiolytic effects following internalization into NPS receptor-expressing neurons. Neuropsychopharmacology.

[47] Dine J., Ionescu I.A., Stepan J., Yen Y.C., Holsboer F., Landgraf R., Eder M., Schmidt U. (2013). Identification of a role for the ventral hippocampus in neuropeptide S-elicited anxiolysis. PLoS ONE.

[48] Bulbul M., Sinen O., Ozkan A., Aslan M.A., Agar A. (2019). Central neuropeptide-S treatment improves neurofunctions of 6-OHDA-induced Parkinsonian rats. Exp. Neurol..

[49] Mochizuki T., Kim J., Sasaki K. (2010). Microinjection of neuropeptide S into the rat ventral tegmental area induces hyperactivity and increases extracellular levels of dopamine metabolites in the nucleus accumbens shell. Peptides.

[50] Si W., Aluisio L., Okamura N., Clark S.D., Fraser I., Sutton S.W., Bonaventure P., Reinscheid R.K. (2010). Neuropeptide S stimulates dopaminergic neurotransmission in the medial prefrontal cortex. J. Neurochem..

[51] Raiteri L., Luccini E., Romei C., Salvadori S., Calo G. (2009). Neuropeptide S selectively inhibits the release of 5-HT and noradrenaline from mouse frontal cortex nerve endings. Br. J. Pharmacol..

[52] Li M.S., Peng Y.L., Jiang J.H., Xue H.X., Wang P., Zhang P.J., Han R.W., Chang M., Wang R. (2015). Neuropeptide S Increases locomotion activity through corticotropin-releasing factor receptor 1 in substantia nigra of mice. Peptides.

[53] Didonet J.J., Cavalcante J.C., Souza L.d.S., Costa M.S., Andre E., Soares-Rachetti V.d.P., Guerrini R., Calo G., Gavioli E.C. (2014). Neuropeptide S counteracts 6-OHDA-induced motor deficits in mice. Behav. Brain Res..

[54] Li W., Gao Y.H., Chang M., Peng Y.L., Yao J., Han R.W., Wang R. (2009). Neuropeptide S inhibits the acquisition and the expression of conditioned place preference to morphine in mice. Peptides.

[55] Enquist J., Ferwerda M., Madhavan A., Hok D., Whistler J.L. (2012). Chronic ethanol potentiates the effect of neuropeptide s in the basolateral amygdala and shows increased anxiolytic and anti-depressive effects. Neuropsychopharmacology.

[56] Cannella N., Kallupi M., Ruggeri B., Ciccocioppo R., Ubaldi M. (2013). The role of the neuropeptide S system in addiction: Focus on its interaction with the CRF and hypocretin/orexin neurotransmission. Prog. Neurobiol..

[57] Cao J., de Lecea L., Ikemoto S. (2011). Intraventricular administration of neuropeptide S has reward-like effects. Eur. J. Pharmacol..

[58] Han R.W., Zhang R.S., Xu H.J., Chang M., Peng Y.L., Wang R. (2013). Neuropeptide S enhances memory and mitigates memory impairment induced by MK801, scopolamine or Abeta(1)(-)(4)(2) in mice novel object and object location recognition tasks. Neuropharmacology.

[59] Gardella E., Romei C., Cavallero A., Trapella C., Fedele E., Raiteri L. (2013). Neuropeptide S inhibits release of 5-HT and glycine in mouse amygdala and frontal/prefrontal cortex through activation of the neuropeptide S receptor. Neurochem. Int..

[60] Okamura N., Garau C., Duangdao D.M., Clark S.D., Jungling K., Pape H.C., Reinscheid R.K. (2011). Neuropeptide S enhances memory during the consolidation phase and interacts with noradrenergic systems in the brain. Neuropsychopharmacology.

[61] Han R.W., Xu H.J., Zhang R.S., Wang P., Chang M., Peng Y.L., Deng K.Y., Wang R. (2014). Neuropeptide S interacts with the basolateral amygdala noradrenergic system in facilitating object recognition memory consolidation. Neurobiol. Learn. Mem..

[62] Ensho T., Nakahara K., Suzuki Y., Murakami N. (2017). Neuropeptide S increases motor activity and thermogenesis in the rat through sympathetic activation. Neuropeptides.

[63] Niimi M. (2006). Centrally administered neuropeptide S activates orexin-containing neurons in the hypothalamus and stimulates feeding in rats. Endocrine.

[64] Grant B.F., Stinson F.S., Dawson D.A., Chou S.P., Dufour M.C., Compton W., Pickering R.P., Kaplan K. (2004). Prevalence and co-occurrence of substance use disorders and independent mood and anxiety disorders: Results from the National Epidemiologic Survey on Alcohol and Related Conditions. Arch. Gen. Psychiatry.

[65] Ersche K.D., Turton A.J., Pradhan S., Bullmore E.T., Robbins T.W. (2010). Drug addiction endophenotypes: Impulsive versus sensation-seeking personality traits. Biol. Psychiatry.

[66] von Diemen L., Bassani D.G., Fuchs S.C., Szobot C.M., Pechansky F. (2008). Impulsivity, age of first alcohol use and substance use disorders among male adolescents: A population based case-control study. Addiction.

[67] Badia-Elder N.E., Henderson A.N., Bertholomey M.L., Dodge N.C., Stewart R.B. (2008). The effects of neuropeptide S on ethanol drinking and other related behaviors in alcohol-preferring and -nonpreferring rats. Alcohol Clin. Exp. Res..

[68] Cannella N., Kallupi M., Li H.W., Stopponi S., Cifani C., Ciccocioppo R., Ubaldi M. (2016). Neuropeptide S differently modulates alcohol-related behaviors in alcohol-preferring and non-preferring rats. Psychopharmacology.

[69] Cifani C., Micioni Di Bonaventura M.V., Cannella N., Fedeli A., Guerrini R., Calo G., Ciccocioppo R., Ubaldi M. (2011). Effect of neuropeptide S receptor antagonists and partial agonists on palatable food consumption in the rat. Peptides.

[70] Beck B., Fernette B., Stricker-Krongrad A. (2005). Peptide S is a novel potent inhibitor of voluntary and fast-induced food intake in rats. Biochem. Biophys. Res. Commun..

[71] Ciccocioppo R., Economidou D., Cippitelli A., Cucculelli M., Ubaldi M., Soverchia L., Lourdusamy A., Massi M. (2006). Genetically selected Marchigian Sardinian alcohol-preferring (msP) rats: An animal model to study the neurobiology of alcoholism. Addict. Biol..

[72] Dine J., Ionescu I.A., Avrabos C., Yen Y.C., Holsboer F., Landgraf R., Schmidt U., Eder M. (2015). Intranasally applied neuropeptide S shifts a high-anxiety electrophysiological endophenotype in the ventral hippocampus towards a “normal”-anxiety one. PLoS ONE.

[73] Lukas M., Neumann I.D. (2012). Nasal application of neuropeptide S reduces anxiety and prolongs memory in rats: Social versus non-social effects. Neuropharmacology.

[74] Vitale G., Filaferro M., Ruggieri V., Pennella S., Frigeri C., Rizzi A., Guerrini R., Calo G. (2008). Anxiolytic-like effect of neuropeptide S in the rat defensive burying. Peptides.

[75] Wegener G., Finger B.C., Elfving B., Keller K., Liebenberg N., Fischer C.W., Singewald N., Slattery D.A., Neumann I.D., Mathe A.A. (2012). Neuropeptide S alters anxiety, but not depression-like behaviour in Flinders Sensitive Line rats: A genetic animal model of depression. Int. J. Neuropsychopharmacol. Off. Sci. J. Coll. Int. Neuropsychopharmacol..

[76] Borruto A.M., Stopponi S., Li H., Weiss F., Roberto M., Ciccocioppo R. (2021). Genetically selected alcohol-preferring msP rats to study alcohol use disorder: Anything lost in translation?. Neuropharmacology.

[77] Stewart R.B., Gatto G.J., Lumeng L., Li T.K., Murphy J.M. (1993). Comparison of alcohol-preferring (P) and nonpreferring (NP) rats on tests of anxiety and for the anxiolytic effects of ethanol. Alcohol.

[78] Domi A., Stopponi S., Domi E., Ciccocioppo R., Cannella N. (2019). Sub-dimensions of Alcohol Use Disorder in Alcohol Preferring and Non-preferring Rats, a Comparative Study. Front. Behav. Neurosci..

[79] Bokstrom K., Balldin J., Langstrom G. (1991). Individual mood profiles in alcohol withdrawal. Alcohol Clin. Exp. Res..

[80] Roelofs S.M. (1985). Hyperventilation, anxiety, craving for alcohol: A subacute alcohol withdrawal syndrome. Alcohol.

[81] Ruggeri B., Braconi S., Cannella N., Kallupi M., Soverchia L., Ciccocioppo R., Ubaldi M. (2010). Neuropeptide S receptor gene expression in alcohol withdrawal and protracted abstinence in postdependent rats. Alcohol Clin. Exp. Res..

[82] Gehlert D.R., Cippitelli A., Thorsell A., Le A.D., Hipskind P.A., Hamdouchi C., Lu J., Hembre E.J., Cramer J., Song M. (2007). 3-(4-Chloro-2-morpholin-4-yl-thiazol-5-yl)-8-(1-ethylpropyl)-2,6-dimethyl-imidazo [1,2-b]pyridazine: A novel brain-penetrant, orally available corticotropin-releasing factor receptor 1 antagonist with efficacy in animal models of alcoholism. J. Neurosci..

[83] Economidou D., Hansson A.C., Weiss F., Terasmaa A., Sommer W.H., Cippitelli A., Fedeli A., Martin-Fardon R., Massi M., Ciccocioppo R. (2008). Dysregulation of nociceptin/orphanin FQ activity in the amygdala is linked to excessive alcohol drinking in the rat. Biol. Psychiatry.

[84] de Guglielmo G., Martin-Fardon R., Teshima K., Ciccocioppo R., Weiss F. (2015). MT-7716, a potent NOP receptor agonist, preferentially reduces ethanol seeking and reinforcement in post-dependent rats. Addict. Biol..

[85] Bifone A., Gozzi A., Cippitelli A., Matzeu A., Domi E., Li H., Scuppa G., Cannella N., Ubaldi M., Weiss F. (2019). phMRI, neurochemical and behavioral responses to psychostimulants distinguishing genetically selected alcohol-preferring from genetically heterogenous rats. Addict. Biol..

[86] Slattery D.A., Naik R.R., Grund T., Yen Y.C., Sartori S.B., Fuchsl A., Finger B.C., Elfving B., Nordemann U., Guerrini R. (2015). Selective breeding for high anxiety introduces a synonymous SNP that increases neuropeptide S receptor activity. J. Neurosci..

[87] Thorsell A., Tapocik J.D., Liu K., Zook M., Bell L., Flanigan M., Patnaik S., Marugan J., Damadzic R., Dehdashti S.J. (2013). A novel brain penetrant NPS receptor antagonist, NCGC00185684, blocks alcohol-induced ERK-phosphorylation in the central amygdala and decreases operant alcohol self-administration in rats. J. Neurosci..

[88] O’Brien C.P., Childress A.R., Ehrman R., Robbins S.J. (1998). Conditioning factors in drug abuse: Can they explain compulsion?. J. Psychopharmacol..

[89] Domi E., Domi A., Adermark L., Heilig M., Augier E. (2021). Neurobiology of alcohol seeking behavior. J. Neurochem..

[90] Moorman D.E., James M.H., Kilroy E.A., Aston-Jones G. (2017). Orexin/hypocretin-1 receptor antagonism reduces ethanol self-administration and reinstatement selectively in highly-motivated rats. Brain Res..

[91] Lawrence A.J., Cowen M.S., Yang H.J., Chen F., Oldfield B. (2006). The orexin system regulates alcohol-seeking in rats. Br. J. Pharmacol..

[92] Schneider E.R., Rada P., Darby R.D., Leibowitz S.F., Hoebel B.G. (2007). Orexigenic peptides and alcohol intake: Differential effects of orexin, galanin, and ghrelin. Alcohol Clin. Exp. Res..

[93] Srinivasan S., Simms J.A., Nielsen C.K., Lieske S.P., Bito-Onon J.J., Yi H., Hopf F.W., Bonci A., Bartlett S.E. (2012). The dual orexin/hypocretin receptor antagonist, almorexant, in the ventral tegmental area attenuates ethanol self-administration. PLoS ONE.

[94] Domi E., Xu L., Patz M., Nordeman A., Augier G., Holm L., Toivainen S., Augier E., Hansson A.C., Heilig M. (2020). Nicotine increases alcohol self-administration in male rats via a mu-opioid mechanism within the mesolimbic pathway. Br. J. Pharmacol..

[95] Domi A., Barbier E., Adermark L., Domi E. (2021). Targeting the Opioid Receptors: A Promising Therapeutic Avenue for Treatment in “Heavy Drinking Smokers”. Alcohol Alcohol..

[96] Okamura N., Habay S.A., Zeng J., Chamberlin A.R., Reinscheid R.K. (2008). Synthesis and pharmacological in vitro and in vivo profile of 3-oxo-1,1-diphenyl-tetrahydro-oxazolo[3,4-a]pyrazine-7-carboxylic acid 4-fluoro-benzylamide (SHA 68), a selective antagonist of the neuropeptide S receptor. J. Pharmacol. Exp. Ther..

[97] Kallupi M., de Guglielmo G., Cannella N., Li H.W., Calo G., Guerrini R., Ubaldi M., Renger J.J., Uebele V.N., Ciccocioppo R. (2013). Hypothalamic neuropeptide S receptor blockade decreases discriminative cue-induced reinstatement of cocaine seeking in the rat. Psychopharmacology.

[98] Melamed J.Y., Zartman A.E., Kett N.R., Gotter A.L., Uebele V.N., Reiss D.R., Condra C.L., Fandozzi C., Lubbers L.S., Rowe B.A. (2010). Synthesis and evaluation of a new series of Neuropeptide S receptor antagonists. Bioorg. Med. Chem. Lett..

[99] Zhang Y., Gilmour B.P., Navarro H.A., Runyon S.P. (2008). Identifying structural features on 1,1-diphenyl-hexahydro-oxazolo[3,4-a]pyrazin-3-ones critical for Neuropeptide S antagonist activity. Bioorg. Med. Chem. Lett..

[100] Schmoutz C.D., Zhang Y., Runyon S.P., Goeders N.E. (2012). Antagonism of the neuropeptide S receptor with RTI-118 decreases cocaine self-administration and cocaine-seeking behavior in rats. Pharmacol. Biochem. Behav..

[101] Bonano J.S., Runyon S.P., Hassler C., Glennon R.A., Stevens Negus S. (2014). Effects of the neuropeptide S receptor antagonist RTI-118 on abuse-related facilitation of intracranial self-stimulation produced by cocaine and methylenedioxypyrovalerone (MDPV) in rats. Eur. J. Pharmacol..

[102] Chou Y.H., Hor C.C., Lee M.T., Lee H.J., Guerrini R., Calo G., Chiou L.C. (2021). Stress induces reinstatement of extinguished cocaine conditioned place preference by a sequential signaling via neuropeptide S, orexin, and endocannabinoid. Addict. Biol..

[103] Tung L.W., Lu G.L., Lee Y.H., Yu L., Lee H.J., Leishman E., Bradshaw H., Hwang L.L., Hung M.S., Mackie K. (2016). Orexins contribute to restraint stress-induced cocaine relapse by endocannabinoid-mediated disinhibition of dopaminergic neurons. Nat. Commun..

[104] Borgland S.L., Ungless M.A., Bonci A. (2010). Convergent actions of orexin/hypocretin and CRF on dopamine neurons: Emerging players in addiction. Brain Res..

[105] Winsky-Sommerer R., Yamanaka A., Diano S., Borok E., Roberts A.J., Sakurai T., Kilduff T.S., Horvath T.L., de Lecea L. (2004). Interaction between the corticotropin-releasing factor system and hypocretins (orexins): A novel circuit mediating stress response. J. Neurosci..

[106] Sakamoto F., Yamada S., Ueta Y. (2004). Centrally administered orexin-A activates corticotropin-releasing factor-containing neurons in the hypothalamic paraventricular nucleus and central amygdaloid nucleus of rats: Possible involvement of central orexins on stress-activated central CRF neurons. Regul. Pept..

[107] Al-Barazanji K.A., Wilson S., Baker J., Jessop D.S., Harbuz M.S. (2001). Central orexin-A activates hypothalamic-pituitary-adrenal axis and stimulates hypothalamic corticotropin releasing factor and arginine vasopressin neurones in conscious rats. J. Neuroendocrinol..

[108] Korotkova T.M., Sergeeva O.A., Eriksson K.S., Haas H.L., Brown R.E. (2003). Excitation of ventral tegmental area dopaminergic and nondopaminergic neurons by orexins/hypocretins. J. Neurosci..

[109] Borgland S.L., Taha S.A., Sarti F., Fields H.L., Bonci A. (2006). Orexin A in the VTA is critical for the induction of synaptic plasticity and behavioral sensitization to cocaine. Neuron.

[110] Korotkova T.M., Brown R.E., Sergeeva O.A., Ponomarenko A.A., Haas H.L. (2006). Effects of arousal- and feeding-related neuropeptides on dopaminergic and GABAergic neurons in the ventral tegmental area of the rat. Eur. J. Neurosci..

[111] Wang B., Shaham Y., Zitzman D., Azari S., Wise R.A., You Z.B. (2005). Cocaine experience establishes control of midbrain glutamate and dopamine by corticotropin-releasing factor: A role in stress-induced relapse to drug seeking. J. Neurosci..

[112] Narita M., Nagumo Y., Hashimoto S., Narita M., Khotib J., Miyatake M., Sakurai T., Yanagisawa M., Nakamachi T., Shioda S. (2006). Direct involvement of orexinergic systems in the activation of the mesolimbic dopamine pathway and related behaviors induced by morphine. J. Neurosci..

[113] Narita M., Nagumo Y., Miyatake M., Ikegami D., Kurahashi K., Suzuki T. (2007). Implication of protein kinase C in the orexin-induced elevation of extracellular dopamine levels and its rewarding effect. Eur. J. Neurosci..

[114] Wang B., You Z.B., Wise R.A. (2009). Reinstatement of cocaine seeking by hypocretin (orexin) in the ventral tegmental area: Independence from the local corticotropin-releasing factor network. Biol. Psychiatry.

[115] Clark S.D., Kenakin T.P., Gertz S., Hassler C., Gay E.A., Langston T.L., Reinscheid R.K., Runyon S.P. (2017). Identification of the first biased NPS receptor agonist that retains anxiolytic and memory promoting effects with reduced levels of locomotor stimulation. Neuropharmacology.

